# Evidence for the Effectiveness of Jungian Psychotherapy: A Review of Empirical Studies

**DOI:** 10.3390/bs3040562

**Published:** 2013-10-24

**Authors:** Christian Roesler

**Affiliations:** 1 Clinical Psychology, Catholic University of Applied Sciences, Karlsstraße 63, 79104 Freiburg, Germany; E-Mail: christian.roesler@kh-freiburg.de; Tel.: +49-761-200-1513; Fax: +49-761-200-1496; 2 Faculty of Psychology, University Basel, Switzerland

**Keywords:** Jungian psychotherapy, empirical research, effectiveness, health care utilization, analytical psychology

## Abstract

Since the 1990s several research projects and empirical studies (process and outcome) on Jungian Psychotherapy have been conducted mainly in Germany and Switzerland. Prospective, naturalistic outcome studies and retrospective studies using standardized instruments and health insurance data as well as several qualitative studies of aspects of the psychotherapeutic process will be summarized. The studies are diligently designed and the results are well applicable to the conditions of outpatient practice. All the studies show significant improvements not only on the level of symptoms and interpersonal problems, but also on the level of personality structure and in every day life conduct. These improvements remain stable after completion of therapy over a period of up to six years. Several studies show further improvements after the end of therapy, an effect which psychoanalysis has always claimed. Health insurance data show that, after Jungian therapy, patients reduce health care utilization to a level even below the average of the total population. Results of several studies show that Jungian treatment moves patients from a level of severe symptoms to a level where one can speak of psychological health. These significant changes are reached by Jungian therapy with an average of 90 sessions, which makes Jungian psychotherapy an effective and cost-effective method. Process studies support Jungian theories on psychodynamics and elements of change in the therapeutic process. So finally, Jungian psychotherapy has reached the point where it can be called an empirically proven, effective method.

## 1. Introduction

Jungian Psychotherapy has long been accused of not giving any empirical proof of its effectiveness. In the early 1990s, the first meta-analyses of empirical studies investigating the effectiveness of psychotherapy were published. Following this, several researchers claimed that there were no studies investigating the effectiveness of Jungian psychotherapy and therefore it should be excluded from the field of psychotherapy. This motivated several Jungian training institutes, namely Zurich, Berlin and San Francisco, to design the first empirical studies in the field of Jungian psychotherapy. Prospective, naturalistic outcome studies and retrospective studies using standardized instruments and health insurance data as well as several qualitative studies of aspects of the psychotherapeutic process were conducted mainly in Germany and Switzerland. The results of these studies will be summarized and critically reviewed in this article. 

In empirical research there is a differentiation between different levels of studies, which is described in the Handbook of psychotherapy and behavior change [1]. The highest level or Gold Standard is the Randomized Controlled Trial (RCT), with an experimental and a control group and a randomized distribution of the participants to the groups. Only RCTs can give proof of the efficacy of a psychotherapy method, which means that the effects on the patients are a result of the method alone (and of no other extra-therapeutic factors; this is equivalent to the term: internal validity). In general, only RCTs are accepted as a proof for the efficacy of the psychotherapy method. In recent years though, there has been a discussion about the validity of RCTs, since their internal validity is high but the external validity, its applicability to every day practice, is low [2]. Several researchers have argued for naturalistic prospective outcome studies which are conducted in every day practice and therefore are much more applicable to real-life conditions. Several of the Jungian studies have used this method. Generally speaking prospective data are more valid than retrospective data. Even though two Jungian studies described below applied a retrospective design, they reached a high validity through careful design.

## 2. Overview of Jungian Empirical Studies

Prospective, naturalistic outcome studies

- Praxisstudie Analytische Langzeittherapie (PAL) Schweiz (Naturalistic study on analytical long-term psychotherapy in Switzerland) [3,4]
- San Francisco Psychotherapy Research Project [5]
- PAP-S Naturalistic study on outpatient psychotherapy in Switzerland [6]

Catamnestic/retrospective studies

- Berlin Jungian Study [7]
- Konstanz Study—A German consumer reports study [8]

Small sample and case studies

- On Jungian sand play therapy [9], psychosomatic disorders [10,11], integration of shadow aspects [12]

Qualitative and process studies

- On complex theory [13], picture interpretation method [14]

### 2.1. Praxisstudie Analytische Langzeittherapie (PAL) Schweiz (Zurich)—Naturalistic Study on Analytical Long-Term Psychotherapy in Switzerland

A group of researchers at the Jung Institute Zurich participated in a larger German study on analytical long-term psychotherapy [4] conducted by the University of Heidelberg and applied the elaborated research design. The design was a naturalistic prospective outcome study, which means that therapists and patients were monitored from the beginning of therapy in the usual everyday practice context (no control group). Twenty-six therapists and their patients, totaling 37 cases, were chosen as representatives for Jungian psychotherapy in Switzerland. Fifty-seven percent of these patients suffered from depressive disorders and with 47% of the patients diagnosed with personality disorders the sample had a considerably high burden of disease. The mean duration of treatment was 35 months with a mean of 90 sessions, which is equivalent to a low-frequency treatment. This was a realistic sample representation for Jungian therapy in Switzerland.

There were three different perspectives applied: researchers, therapists and the patients themselves. On each level a set of objective and self-evaluation measures were used.

#### 2.1.1. Researchers

Operationalized psychodynamic diagnostics (OPD), Jungian adaptation [15]: OPD is a complex set of dimensions systemizing and operationalizing psychoanalytic diagnostic interviews, e.g., types of unconscious conflicts, maturity of personality (ego) structure, *etc.* This was adapted to Jungian theoretical concepts (e.g., complex theory) for the PAL-study.

Psychodynamic focuses (two interviews): focus means the main unconscious conflicts (e.g., attachment *vs.* autonomy) identified via OPD that are treated in analysis. 

Changes in personality structure (Heidelberger Umstrukturierungsskala): measures changes in the maturity levels of personality/ego functions identified via OPD.

Therapeutic alliance and transference (SGRT: spontane gefühlshafte Reaktion, TAB: therapeutische Arbeitsbeziehung): external rating of quality and character of the therapeutic relationship, working alliance and transference.

Interpersonal problems (Interpersonal Problems Inventory, IIP)

Changes in life conduct (research interview)

#### 2.1.2. Therapists

Physical and psychological symptoms:

Severity of symptoms score (Beeinträchtigungsschwereskala, BSS): measures the impairment the patient suffers through the symptoms.

Status and process ratings:

ICD10 (International Classification of Diseases, chapter 10: psychological disorders)-diagnosis

#### 2.1.3. Patients

Psychological and interpersonal symptoms: Symptom Checklist 90 Revised Version (SCL-90-R), the most widely used clinical measure in psychotherapy research; psychological/social/communicative competencies measure (PSKB-Se-R); 

Interpersonal Problems Inventory (IIP); 

Trier Personality Inventory (TPF);

Health insurance data (use of health care services, visits to primary care physicians, days in hospital *etc.*)

#### 2.1.4. Results

(The term effect size describes the impact the therapy method has in moving the patient sample from an area of disorder to an area of normal health.)

Researchers:

- Positive restructuring of patient’s personality, effect size: 0,94.
- Positive changes in everyday life, very high effect size: 1,48.

Therapists:

- Global rating of results positive or very positive for 75% of therapies
- Cost-effectiveness good, very good or maximum for 55% of therapies

Patients:

- Global Severity Index (the global measure of the SCL-90-R) reduced highly significant, very high effect size: 1,31, normal level at end of therapy
- Significant reduction of interpersonal problems (IIP), medium effect size
- Rating of results over 90% positive, very positive or maximum
- Cost-effectiveness 80% good, very good or maximum, 20% satisfying

All these reported results were significant (5%-level) or highly significant (1%-level).

#### 2.1.5. Follow-up

All results remained stable after one year and three years. An interesting point is that there are findings for further positive effects between the end of therapy and follow-up, which would mean that some effects of the therapy show only after the end of therapy; this is an effect that psychoanalysis has always claimed. The use of healthcare services was already low during the course of therapy and remained on a low level until the follow-up.

This study could give proof for very positive effects of Jungian psychotherapy in a prospective design that remains stable over three years after the end of therapy. Jungian therapy leads not only to a significant reduction of symptoms and of interpersonal and other problems, but also to a restructuring of the personality with the effect that the patients can deal with upcoming problems much better after the end of therapy. The satisfaction of the patients with the results was extremely high even though most of the patients had to pay for their therapy themselves. The limitation of the study is the lack of a control group which poses the question whether the sample may be an especially highly motivated group of patients, even though the severity of symptoms was high and representative for the population of patients in Switzerland.

### 2.2. San Francisco Psychotherapy Research Project

Originally this study conducted by the San Francisco Jung Institute was designed as a prospective outcome study with four points of measurement (start of therapy, end of therapy, one-year and five-year follow-up). In many aspects the design of the San Francisco psychotherapy research project is similar to that of the Zürich study. The measures applied were: SCL-90-R; IIP, GAF (Global Assessment of Functioning Scale, rated by external experts); an additional instrument designed by the Institute asking for demographic data, therapy motivation and subjective experience with the therapy; the therapists had an instrument also designed by the Institute called “Portrait of my practice” (POMP), which asked for structural aspects as well as the personal style and background of the therapist. The participants of the study were patients of the outpatient clinic of the San Francisco Jung Institute; of 100 patients in the clinic, 57 participated in the study. The participating therapists were 23 professional analysts of the Institute as well as 17 candidates in training and seven psychology interns. 

Because of the low participation of analysts from the Institute, the project had to be terminated early. Because of these problems, the original design had to be collapsed into a one-group pretest-posttest-design. This included 39 of the original 57 patients and only part of these completed follow-ups. The internal validity of the study could not be secured and the statistical results have to be interpreted on that background. Only data from the start and end of therapy could be compared. Bearing these limitations in mind, the study still points in the direction of proving effectiveness of Jungian therapy; there were significant reductions in SCL-90-R and IIP. 

### 2.3. Berlin Catamnestic Study

In the early 1990s the Empirical Psychotherapy Research Group in Analytical Psychology Berlin conducted a nationwide catamnestic, retrospective study [16,17]. Former patients of Jungian psychotherapies were asked to participate and were tested via questionnaires and interview. All members of the German Society for Analytical Psychology (DGAP) were asked to participate in this retrospective study: 78% responded, 24.6% participated. In retrospective studies there is always the danger of a bias in the sense that only successful patients (or therapists) are willing to participate, which would give no realistic picture of the results. So the reasons for refusal to participate were documented and no bias was found. The participating therapists documented all cases terminated in 1987/1988 and gave a comprehensive evaluation of the success of therapy. In Germany, psychotherapy is financed quite generously by the health insurance companies (up to 300 hours of analysis); at the beginning of therapy the therapist has to apply for financing. These applications contain numerous data about the health state and symptoms of the patient, the personality, the social context, the psychodynamics and diagnosis. This information is stored by the health insurances for decades and the Berlin study made use of these data. Additionally, other health insurance data about the patients could be used, for example, their use of healthcare services, days in hospital, *etc.* The distribution of symptoms and their severity in the sample were as follows: 46% affective disorders, 24% other neurotic and psychosomatic disorders, and 17% personality disorders. 

The problem with catamnestic studies is the risk of biases through selection effects, but these were tested in the study: of 353 documented cases 111 participated in the study; a bias was found concerning the number of therapy drop-outs which was higher in the sample than in the population; apart from that the sample was representative for the population. The mean duration of treatment was 162 sessions with a frequency of one to two sessions per week.

**Figure 1:**
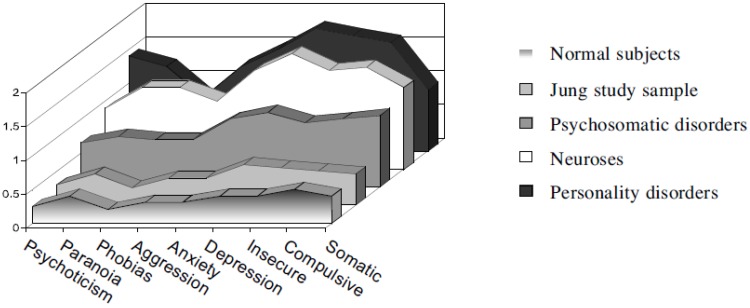
Mean SCL-90-R measures on follow-up compared to standardization samples (Figure taken from Keller *et al.* 1997 with kind permission from the author).

Results: of 60.4% of patients reporting their well-being as very poor (severe set of diagnoses) prior to therapy, 86.6% rated their global well-being at follow-up as very good, good or moderate (well-adjusted close to normal reference group on all scales of psychopathology). Six years after the termination of treatment 70%–94% reported good to very good improvements in: psychological distress, general well-being, life satisfaction, job performance, partner and family relations, and social functioning. The global health state of 88% could be described as “normal health”. Patients were better off than any of the clinical groups with which they shared diagnoses prior to therapy. Regarding the SCL-90-R Jungian therapy could move the sample of severely disturbed patients close to a standardization sample of normal subjects where one can speak of psychological health (see Figure 1 below). 

**Figure 2:**
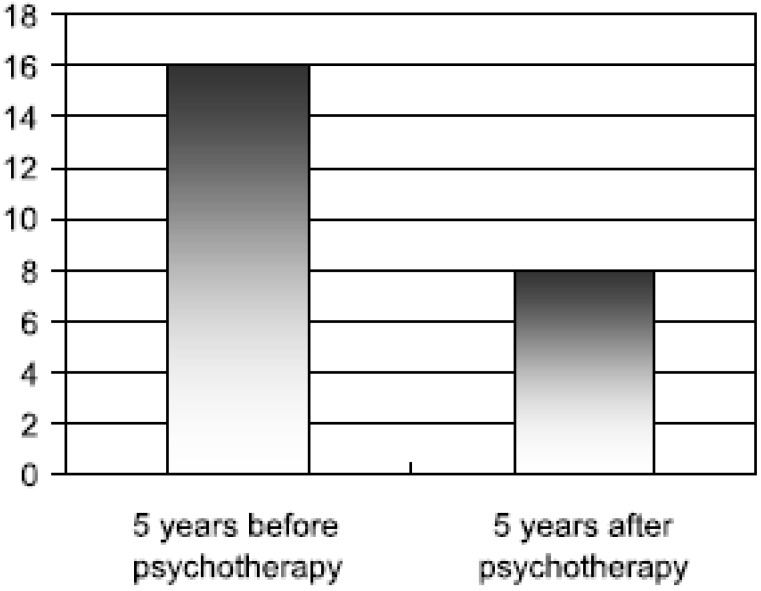
Mean number of days lost per annum due to sickness (Figure taken from Keller *et al.* 1997 with kind permission from the author).

**Figure 3:**
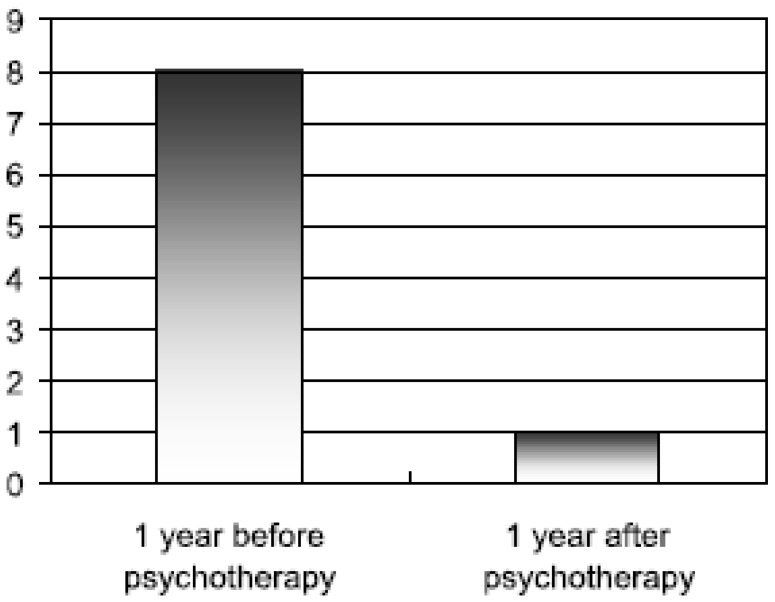
Mean number of days of hospitalization (Figure taken from Keller *et al.* 1997 with kind permission from the author).

All of these results were statistically highly significant. There was also a significant reduction of health insurance claims: the mean number of days lost due to sickness, the mean number of days of hospitalization, the intake of psychotropic drugs and the number of visits to primary care physicians were all significantly reduced even below the level of the average German member of the health insurance system (see Figure 2 and Figure 3 below). 

Other interesting findings are seen in the relationship between improvement and treatment length and again there are indicators for further improvements after termination of therapy (between post- and follow-up). 

Summarizing the results it can be said that there was not only a high satisfaction of the patients with the Jungian psychotherapy but there was also a reduction in symptoms, which moved the patients into the area of normal health. The effects of psychotherapy were long-lasting and touched all areas of the life of the patients so that even the use of healthcare services was so drastically reduced that Jungian therapy was also cost-effective in the long run. These results have to be interpreted against the background of limitations of the design even though the study made great efforts to control biases and secure the representativeness of the sample.

### 2.4. Konstanz-Studie—A German replication of Seligman’s Consumer Reports Study

The study conducted in Constance/Germany is a replication of the famous Consumer Reports Study done by Seligman [8] applied to therapies from several psychodynamic schools and in its design comparable to the above-mentioned Berlin study. Ninety psychotherapists distributed 979 questionnaires to former patients of whom 66% participated in the study. There were no systematic biases found in the sample. About a fifth of the participating therapists had a Jungian background and it could be shown that there are no systematic differences between this subgroup and the overall sample so that the study is representative for psychoanalytic practice in Germany in general and for Jungian psychotherapy.

The results are very much comparable to those of the above-mentioned Berlin study, in all dimensions the study found significant benefits in health and well-being. There were again significant changes between end of therapy and follow-up. As in the Berlin study health insurance data were used and there was found to be a highly significant reduction in health utilization parameters. All of these, results remained stable in a six-year follow-up. A special aspect of this study is the carrying out of a cost-benefit computation: there were significant savings accrued as a result of individual and group psychotherapy in the first two years after therapy (see Table 1 below). These were significantly higher in relation to the severity of the health status of the patient at the beginning of therapy.

**Table 1:** Savings accrued as a result of individual and group psychotherapy in the first two years after completion of therapy (table taken from Breyer *et al.* 1997 with kind permission from the authors).

	Individual therapy	Group therapy
Savings through expected reduction in health care events (doctor visits, days sickness, days in hospital)	8,477.80 DM	14,330.00 DM
Costs of treatment	33,235.00 DM	4,305.00 DM
Savings/costs ratio	0.255 : 1	3.32 : 1

As this study is a retrospective study the results have to be interpreted against the background of risk of biases but these were controlled for as far as possible.

### 2.5. Praxisstudie Ambulante Psychotherapie Schweiz (PAP-S)—Practice Study Outpatient Psychotherapy Switzerland

This study realized a quasi-experimental design, which is the highest level of all the studies described here. The design is comparable to that of the Zurich Jungian study but additionally it has a parallel control group. In Switzerland, all of the different psychotherapeutic schools are organized in the Charta for Psychotherapy and this was the organizer of the PAP-study. The choice of measures applied followed the recommendations given by the Society for Psychotherapy Research and includes outcome as well as process variables. Measures for the patients included: self rating of therapy outcome (Outcome Questionnaire OQ 45), symptoms (Brief Symptom Inventory BSI—the short version of SCL-90-R), depression (Beck Depression Inventory BDI), Sense of Coherence (SOC-9), congruence (K-INK—means self congruence as defined by Carl Rogers), and therapy motivation (FMP). For the researchers: Standardized Clinical Interview for DSM (SKID), Global Assessment of Functioning Individual (GAF) and Relationships (GARF), and Operationalized Psychodynamic Diagnostics (OPD). The study ran seven years (2006–2012) including therapies and follow-up.

The participating psychotherapists mainly utilized psychodynamic and experiential approaches. The problem was that even though the Swiss Jungian Association paid the largest part of the study there were only four Jungian cases participating in the study, which is far too small a number to compute a result for Jungian therapy alone. Even though all the Swiss Jungians were asked to participate, the majority was reluctant. 

Nevertheless, the study produced some interesting findings. Generally all the participating schools were successful in improving the health status of the patients significantly and effectively. A part of the study consisted in describing the interventions in detail that are applied by the different schools. In the study, therapies were videotaped and external raters evaluated which of the described interventions were practically applied. This may be the most interesting finding of the whole study: in every school the majority of interventions applied was not school-specific but either general or stemming from a different school. Only about 15% of the interventions came from the specific background of the therapist. This is a finding that other studies produced that were also investigating the question of the school specificity of interventions applied. Already in the so-called generic model of psychotherapy by Orlinsky [18] it was assumed that there are common factors applied in all schools of psychotherapy that make the greatest part of the impact of psychotherapy. Keller [19] has compared the common factors model with the central interventions used in Jungian psychotherapy and has found many parallels. This of course automatically puts the question whether there even is specificity in the practical therapeutic work of Jungian therapists and what that would be. After so many studies certified the so-called “Dodo-verdict” showing that all schools seem to be equally effective, the current trend in psychotherapy research is to look at differences between therapists and investigate what they actually do when they “do therapy”. 

### 2.6. Small Sample Studies, Case Studies and Qualitative Process Research

At the Pontifica Universidade Catolica de Sao Paulo, Brazil, there has been a clinical psychology department with an explicitly Jungian orientation for many years. In the Master and Doctoral program, a couple of empirical research papers were produced. Just a few of them will be summarized now. A group of papers investigated the effectiveness of sand play therapy and other explicitly Jungian interventions in different psychosomatic diseases [9,10,11]. In general, the application of Jungian methods, especially that of sand play therapy, had a very positive effect on the well-being of the patients and in some cases even lead to remission of the physical symptoms. Additionally the papers could show that the psychodynamics behind the psychosomatic disorder clearly influenced the symbolism in the sand pictures and that there was a parallel development between the symbols in sand play therapy and improvements in the well-being of the patients.

Other investigations attempted to catch other aspects of Jungian psychotherapy interventions and make them accessible for empirical research. Krapp [14] has developed a systematic method for interpretation of pictures from psychotherapeutic processes. Kleeberg [12] investigated the development of shadow symbols in several psychotherapy processes and could show that the unconscious symbols pictured important aspects of the therapeutic relationship. In a single case study on complex theory [13], Heisig investigated the development of complexes in the course of an analytical psychotherapy and could show that in the first phase of the therapy the complexes were reproduced in the transference relationship, whereas towards the end of therapy the ego complex could slowly separate from other complex patterns which can be understood as a process of ego strengthening.

## 3. Discussion

When we put the studies on Jungian therapy in the matrix of evidence-based therapy we get the following:

Level I (randomized controlled trials): no studies
Level II (quasi-experimental studies; prospective naturalistic outcome studies): PAL-Study, San Francisco Research Project (with limitations); PAP-S Study (with control group)
Level III (retrospective studies): Berlin Jungian Study and Constance Study with very high methodological level
Level IV (case studies *etc.*): positive effects through sand play therapy, in psychosomatic disorders *etc.*

As there are, up to now, no level I studies (RCTs) there is no proof of efficacy of Jungian psychotherapy, but the effectiveness of Jungian psychotherapy is now empirically proven on the base of the above-mentioned studies; the same can be said for the cost-effectiveness. As most of the studies are naturalistic designs it can be assumed that they give a realistic picture of Jungian therapy in every day practice. All of the studies report positive effects in a wide variety of disorders with good or very good effect sizes on: symptom reduction, well being, interpersonal problems, change of personality structure, reduction of health care utilization, and changes in everyday life conduct. All of these effects are stable in follow-ups up to six years after therapy. There are even further positive changes between termination and follow-up. With an average of only 90 sessions, Jungian therapy is a very time- and cost-effective form of psychodynamic psychotherapy. All the studies realized a high methodological standard with objective measures, different research perspectives (patient, therapist, researcher), and control of biases. The most convincing result concerning the effectiveness of Jungian psychotherapy in the overview of all studies is that their results all point in the same direction even though they had quite different patient samples and applied very different methodologies. Nevertheless, the efficacy of Jungian psychotherapy is still to be proven in a randomized controlled trial design.

A very interesting point is that in all the studies that realized a follow-up, further improvements were found after the end of therapy. In the theoretical model of analytical psychology it was always assumed that some effects would emerge only after the therapeutic relationship has ended. The empirical studies described here give proof of this assumption. This can also be interpreted as evidence for the fact that analytical psychotherapy not only changes symptoms but also the structure of personality in a deeper sense which leads to a better adaptation to life contexts and relationships but needs some time to unfold. Therefore, future research should always include a follow-up to catch this effect of analytical psychotherapy.

On the other hand, the overview of the studies indicates some recurrent problems. We have to note that in all studies 10%–20% of patients did not profit from Jungian therapy. This is a common finding also in other studies investigating other schools of psychotherapy. Nevertheless, this should be subject to further research aiming at finding markers for personalities expected to profit from Jungian psychotherapy.

Another severe problem that comes to light in the overview of the studies is the fact that Jungian analysts tend to be very reluctant in participating in empirical studies to the extent that leads almost to the breakdown of studies. From the beginning there were difficulties in recruiting enough practicing analysts to participate in the studies, which is still a problem today as can be seen in the latest example, the PAP-study Switzerland. One of the main arguments against participating in empirical studies was the assumption that the research process would interrupt or at least influence the analytic process and the therapeutic relationship in an unfavorable way. Also it was argued that empirical instruments would never be able to catch the complexity of the analytic process. From my point of view these critical positions are based on false ideas about the research process, its capacities and its limitations. Of course any research design to investigate psychotherapy has its limitations and can only analyze certain aspects of the complex interactions taking place in the process of psychotherapy. However, empirical research methods offer the possibility to get an insight into the psychotherapeutic work and its effects from a more objective position. We have to consider that the perspective of practicing psychotherapists on their own processes is, and has to be, mainly subjective and is subject to interpretation and also to the possibility of error. On the other hand, empirical research can never claim to tell the whole truth about psychotherapy. We also have to consider that the work of psychotherapy has a major impact on the lives of the clients and therefore it is an ethical requirement to install quality management processes of which psychotherapy effectiveness research is one.

From my point of view this should be a point of discussion in the Jungian community. At least it can be said now that the point that was often made from critics of empirical research in the Jungian community—that empirical methods would interfere with the special situation of the analytical relationship—has been falsified by the above studies: in no study there was any hint of a negative interference into the psychotherapeutic process; some studies made great efforts to adapt or even develop research measures which catch aspects specific to the Jungian background, for example, changes in personality or the adaptation of psychodynamic diagnostics [15]. On the other hand, Jungian psychotherapy can now offer empirical results about the effectiveness of its method and is no longer subject to the critique that the method is not effective or empirically proven. For a more detailed description and discussion of research in Jungian psychology see the German publication by Roesler [20].

## 4. Prospects: Currently Ongoing Studies in Germany

The German Association of Analytical Psychology has formed a research platform (www.cgjung.de/forum), which is currently planning to conduct several studies in the field of Jungian psychotherapy. The training institutes are working on an agreement that future training candidates will have to apply a set of empirical measures (symptoms, life satisfaction, Operationalized Psychodynamic Diagnostics) to their training cases in order to form a database and to make ongoing quality management possible. In the long run this aims at creating a more open attitude to empirical research in the coming generations of Jungian analysts. On the other hand this process aims at stabilizing the currently comfortable position Jungian therapy has in the German healthcare system for the future, by delivering empirical results on the effectiveness of the methods and applying standard quality management processes.

Structural dream analysis: The author has developed a narratological qualitative research method for analyzing dream series from analytical psychotherapies and extracting the core process of change in the course of the psychotherapy [21]. At the moment a number of dream series from Jungian psychotherapy processes are being analyzed using this method in a research project at the University of Basel, Switzerland. After the Structural Analysis of a dream series is completed, the results are compared with the report from the psychotherapist about the process of the therapy. This project aims at building a corpus of cases, which would make it possible in the long run to show that the unconscious produces therapeutic change via dreams in the course of an analytic therapy.

In another research project, a documentation scheme for systematic documentation of synchronistic events taking place in psychotherapy is applied [22]. This documentation scheme is now distributed in the German Jung Association and practicing analysts are invited to document relevant events to build up a corpus of cases, which will be subject to further analysis. This project aims at building an empirically based theory of synchronicity in psychotherapy.

In general, these projects and attempts aim at generating a more research open attitude in the Jungian community and a more evidence-based foundation of the theoretical models of Analytical Psychology.
